# Ultra-quick dynamics and acrobatics of viscous marbles

**DOI:** 10.1038/s41467-026-69128-2

**Published:** 2026-05-13

**Authors:** Auriane Huyghues Despointes, Yui Takai, Shoko Ii, Timothée Mouterde, David Quéré

**Affiliations:** 1 Physique et Mécanique des Milieux Hétérogènes, UMR 7636 du CNRS,PSL Research University, ESPCI, Paris, France; 2 Department of Mechanical Engineering, School of Engineering, The University of Tokyo, Tokyo, Japan

**Keywords:** Fluid dynamics, Applied physics

## Abstract

Non-wetting viscous drops are unusually quick compared to wetting ones, owing to the conjunction of non-wetting (which minimizes the contact with the substrate) and rotation (which minimizes the dissipation inside the liquid). Here, we report the existence of a threshold in driving force above which the speed of these objects increases about tenfold compared to previous models and experiments. We find that this effect is due to a dynamic reduction of the contact, that minimizes even more the interaction with the substrate but can also render the dynamics non-stationary – a consequence of the geometrical transformations of fast revolving drops. Paradoxically, viscosity enables this regime by impeding rapid contact equilibration, thereby reducing substrate interactions in fast-moving drops.

## Abstract (web summary)

The dynamics of nonwetting viscous droplets, such as liquid marbles, are significantly different from those of wetting droplets, leading to unexpected behaviors in their movement on inclined surfaces. This study reveals that these marbles can achieve speeds up to ten times greater than previously predicted when subjected to sufficient driving forces, due to a dynamic reduction in contact with the Perfect. substrate, highlighting their potential for applications requiring enhanced mobility.

## Introduction

While viscous droplets are notoriously sluggish on inclines^1–3^, the picture is markedly different when liquids non-wet their substrate. Different effects contribute to the mobility: firstly, obtuse contact angles minimize dissipation close to contact lines^2,3^; secondly, non-wetting drops have a reduced interface with solids, which lowers even more friction^4^; thirdly, viscous droplets rotate so that dissipation is limited to the (reduced) contact zone^4^. As an archetype of non-wetting drops, we consider here liquid marbles, namely drops covered by a hydrophobic powder that prevents direct connection with the substrate, whatever the nature of this substrate^5^. The flourishing research about these objects has mainly focused on their fabrication, versatility and multidisciplinary applications, as reported in the recent review articles by Tenjimbayashi and Tong^6,7^, but the question of their mobility has been much less studied—this is the object of this paper.

**Fig. 1:**
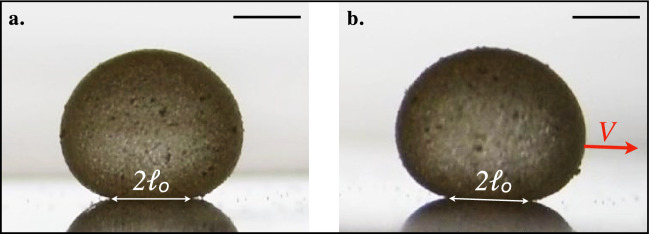
Liquid marbles. **a** A liquid marble is a drop coated by a layer of grains that prevents the liquid from contacting the solid on which it is placed. The non-wetting drop (radius *R* = 1.3 mm) is deformed by gravity close to the solid, and we denote *ℓ*_o_ as the radius of this “contact”. The bar indicates 1 mm. **b** If the substrate is slightly tilted (here by 2°), the marble (here filled with glycerol, viscosity η ≈ 1000 mPa.s) rolls at a speed *V* of 3 mm/s, but it keeps its static shape.

Figure 1a shows a millimetric marble placed on smooth steel: the object is quasi-spherical, and it meets the subjacent solid with an angle that cannot be distinguished from 180°^8^. However, it develops a gravity-driven contact with the substrate, whose size *ℓ*_o_ (here 0.6 mm) is significantly smaller than its mean radius *R* (here 1.3 mm), the hallmark of non-wetting. If we slightly tilt the substrate (Fig. 1b), the drop moves, yet with the same shape, as assumed by Mahadevan and Pomeau (MP) to calculate its speed *V* ^4^. Here we describe the dynamics of viscous marbles on inclines whose tilt is varied by a large amount, so as to generate a large interval of driving forces and speeds. The conceptually important regime at small tilt (up to 8°) has been discussed theoretically and experimentally^4,5,9,10^. But we shall see that marbles at larger tilts are much quicker than expected—a valuable property in applications involving viscous drops, where it is often desired to promote mobility.

Viscous marbles are made by gently shaking drops of glycerol on a bed of lycopodium spores purchased from Fluka^5^. These porous hydrophobic grains with a diameter of ∼30 μm^11^ stick to the surface of the drop, yet without deeply modifying its surface tension γ ≈ 63 mN/m and density ρ ≈ 1260 kg/m^3^ ^5,12^. At room temperature (∼24 °C), the resulting marbles have a viscosity η ≈ 1000 mPa.s, that is, three orders of magnitude larger than that of water, but they remain Newtonian at all shears tested in this study^13^. Their volume Ω is between 1 and 20 μL, which makes their radius *R* always smaller than the capillary length *a* = (γ/ρ*g*)^1/2^ (about 2.5 mm for *g* = 9.8 m/s^2^), the condition for restricting the effect of gravity on drop shapes to the flattening of their base.

## Results

**Fig. 2:**
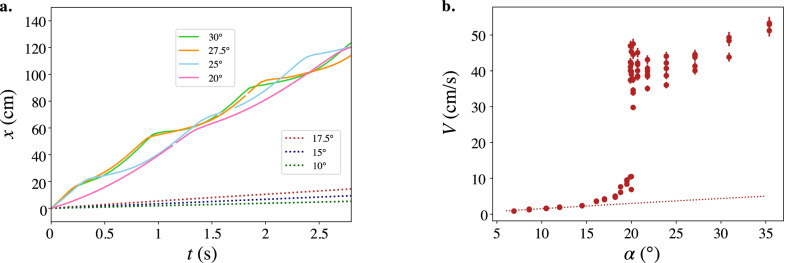
Dynamics of viscous marbles. **a** Position *x* as a function of time *t* of glycerol marbles (Ω = 18 µL) on steel strips tilted by an angle α. At moderate angles (α < 18°), the descent is stationary: the marble moves at a constant speed d*x*/d*t* of a few centimeters per second. When α exceeds 20°, the dynamics become non-stationary (wavy pattern), and the velocity d*x*/d*t* oscillates between 30 and 80 cm/s within each cycle. See also Movie 1a, b in the Supplementary Information (SI). **b** Mean marble velocity *V* as a function of α. Dots show the Mahadevan-Pomeau (MP) speed *V*_o_ = γα*a*/η*R*^4^, where the numerical coefficient of 1 provides the best fit to the data (γ is the surface tension and *a* the capill*a*ry length). Data tends to the MP law at low α, but it strongly deviates between 15° and 20°, where speed can become ten times higher than predicted; at larger angles, data is scattered, and *V* slowly increases with α. Error bars indicate the uncertainty in the initial and final positions of the drop; when not visible, they are smaller than the symbol size.

Marbles are then placed on long strips (several meters) of steel with nanometric roughness (mirror quality), and the strips are inclined at an angle α ranging from a few degrees to a few tens of degrees. The tilt is measured using a digital inclinometer, with an accuracy of ±0.2°. Using high-speed images shot from the side at a rate of 300 to 8000 frames per second, we extract the drop position *x* as a function of time *t*, which we plot in Fig. 2a for slopes α of 10° to 30° and for Ω = 18 μL. We deduce from these plots an average speed of descent *V*, which we display in Fig. 2b as a function of α.

Contrasting with Galileo’s solid marbles, viscosity makes the initial phase of acceleration invisible at the metric scale of the experiment. At low tilt (α < 18°, dotted lines), the marble position *x* linearly varies with time *t* (Fig. 2a), which provides a constant velocity *V* of a few centimeters per second (Fig. 2b). In contrast, what happens for α > 20° is dramatic: firstly, the curves become wavy, indicating that the velocity oscillates between low and high values, yet in a regular way: variations are roughly periodic, with a period that decreases when tilting more the substrate; secondly, the mean velocity jumps by approximately one order of magnitude, so that it reaches values of order 50 cm/s (Fig. 2b).

The small slope limit was modeled by Mahadevan and Pomeau for viscous droplets with *R* < *a*^4^. Firstly, the size *ℓ*_o_ of the contact results from a balance between Laplace pressure and gravitational stress that respectively scale as γ*/R* and ρ*gR*^*3*^*/ℓ*_o_^*2*^. Hence, we get *ℓ*_o_ ~ *R*^2^/*a*, which is quadratic in *R* instead of linear for more wetting drops. Secondly, marbles at small Reynolds numbers rotate like solid spheres, except in the contact zone, due to the no-slip condition. Viscous dissipation being localized in this zone, the viscous torque scales as (η*V/R*)*ℓ*_o_^3^, assuming that contact keeps its static size *ℓ*_o_ (Fig. 1b). Balancing it with the gravitational torque ρ*g*α*R*^4^ yields the MP descent velocity *V*_o_ ~ (γα/η) (*a*/*R*), where the missing numerical coefficient was found by Schnitzer et al. to be of order unity^10^. The surprising dependency of *V* in *R* (smaller drops are quicker) was confirmed by Aussillous on slopes α of a few degrees^5^. In addition, this result implies that the time needed to reach the velocity *V*, starting from rest, scales as ρ*a*^3^/η*R*. It is independent of the velocity and of order 10 ms, a duration much smaller than the timescale of our experiments.

Drawn with dots in Fig. 2b with a numerical coefficient of 1, the MP law asymptotically fits data at small tilt. However, it underestimates the speed as α increases, *V* being three times larger than predicted for α ≈ 20°, before jumping by a new factor ~4 around this critical value. The speed is then as high as ~50 cm/s, comparable to that of non-wetting water “pearls” (a few m/s^14^) despite a factor 1000 in the viscosity. At larger tilts (beyond 20°), data is scattered owing to the waviness of the function *x*(*t*), but it still exhibits a weak increase. A main feature in Fig. 2b is its criticality, with a tenfold increase of speed compared to the MP prediction while the driving force was only augmented by 30%.

As seen in Fig. 2b, the mean speed of marbles lies between 1 cm/s and 50 cm/s, with corresponding Reynolds numbers Re = ρ*VR*/η between 0.01 and 0.5, hence smaller than unity: drops rotate and keep a viscous friction with their substrate—two key ingredients in the MP model^4^. This model also assumes small capillary numbers Ca  = η*V*/γ, such that surface tension resists deformations induced by viscous friction. While this number is around 0.15 for *V* = 1 cm/s, it exceeds unity when the velocity is above 6 cm/s, suggesting that the liquid might abandon its quasi-static shape at moderate slopes. Since viscous dissipation in the MP model concentrates within the contact region, we focus on this region and extract its radius *ℓ* from high-speed movies at all marble velocities *V* (Fig. S1 in the SI).

**Fig. 3:**
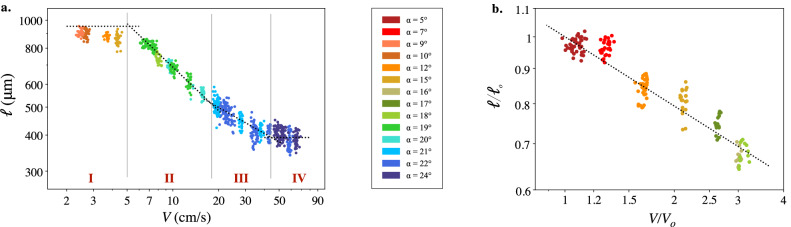
Contact of dynamical marbles. **a** Contact radius *ℓ* as a function of the instantaneous speed *V*, in both stationary and non-stationary situations. The marbles' volume and viscosity are Ω = 18 μL and η = 1000 mPa.s, and colors indicate α (9°<α <24°). We identify four regimes of contact, I to IV, all discussed in the paper, and dots show lines with respective slopes 0, −1/2, −3/10, and 0, and prefactors 1, 0.7, 0.7, and 0.6. The first plateau shows the radius *ℓ*_o_ of the static MP contact, from which data deviates more and more as *V* increases: the contact is squeezed by motion, which qualitatively explains why marbles can be quicker and quicker. **b** Contact *ℓ* as a function of the speed *V*, respectively normalized by the static value *ℓ*_o_ and the MP speed *V*_o_ for Ω = 10 μL and η = 650 mPa.s (water/glycerol mixture). Data are obtained in regime I, where all dynamics are stationary (constant marble velocity), for 5° <α <18°. The straight dotted line has a slope – 1/3.

In Fig. 3a, we plot *ℓ* as a function of *V* for instantaneous speeds ranging from ~3 to ~70 cm/s, corresponding to slopes α between 9° and 24° that encapsulate all situations studied in the paper. Each color stands for a fixed α, and the variation of the velocity along the trajectory at large α (Fig. 2a) explains why a given angle can generate several bunches of data. We did at least ten experiments per bunch, which yields the dispersity. Figure 3a reveals the dynamical nature of the contact when the speed is larger than a few centimeters per second. The radius *ℓ* decreases with *V*, starting from the static plateau value *ℓ*_o_ down to a second plateau where *ℓ* has been divided by ~2.5. This implies a factor of order 10 in the contact volume *ℓ*^3^, thus large enough to generate the jump in velocity seen in Fig. 2b. We place four numbers in the figure, from I to IV, for evidencing the various regimes of contact further described in the paper.

Regime I is the stationary regime of descent (constant speed, straight lines in Fig. 2a), only observed at moderate α (5° < α < 18°). At low speed, the contact size *ℓ* asymptotically tends toward its static value *ℓ*_o_ corresponding to the highest plateau in the figure. However, *ℓ* gradually deviates from *ℓ*_o_, as highlighted in Fig. 3b, where *ℓ*/*ℓ*_o_ is plotted in regime I as a function of the speed *V* normalized by the MP velocity *V*_o_. *ℓ* decreases with *V*, a lowering that reaches 30%. The sensitivity of the speed in contact size can be understood by generalizing the MP model to dynamical situations. Writing the torque balance with a dynamic contact *ℓ*, (η*V/R*)*ℓ*^3^ ∼ ρ*g*α*R*^4^, yields a speed *V* ∼ ρ*g*α*R*^5^/η*ℓ*^3^ that implies that marbles will be much quicker if their contact is smaller. This law, *ℓ*/*ℓ*_o_ ∼ (*V/V*_o_)^−1/3^, is tested in Fig. 3b where the dots with slope −1/3 indeed nicely describe the data.

## Discussion

**Fig. 4:**
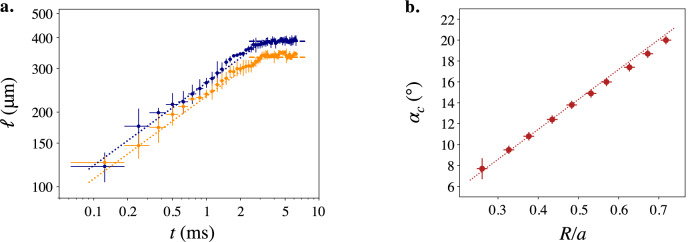
Contact dynamics and consequences. **a** Evolution of the contact radius *ℓ* of a viscous marble that meets a flat solid at *t* = 0. The marble is made of glycerol, and its volume Ω is either 3.4 μL (orange data) or 4.5 μL (blue data). Dotted lines have a slope of 1/3, the spreading exponent predicted by our scaling model, in which we introduce a numerical factor 1.1 to fit the data. At “long” time, *ℓ* reaches the static radius *ℓ*_o_ of a non-wetting drop with volume Ω (dashed plateaus). Horizontal error bars arise from the acquisition rate, whereas vertical error bars reflect dispersion due to limitations in contact definition. **b** Critical angle α_c_ above which the velocity of a glycerol marble “diverges”. α_c_ is linear in *R*/*a*, and the prefactor chosen for the fit is 0.5. Error bars correspond to the dispersion of the results.

We now discuss the physical origin of the reduction of contact and why the effect is stronger at higher speeds (regimes II, III, and IV in Fig. 3a). We understand it as a consequence of rotation. A revolving drop constantly creates a new flat base, which is not instantaneous. If we denote τ as the time needed by a spherical drop to establish an MP contact *ℓ*_o_, we anticipate two cases. If τ is shorter than *ℓ*_o_/*V*, the “residence time” of the MP contact on the solid, this contact can be quasi-static, with size *ℓ*_o_. In contrast, for τ > *ℓ*_o_/*V*, the contact becomes dynamical, with a size *ℓ*(*V*) smaller than *ℓ*_o_. In order to determine τ, we study how a viscous marble “spreads” on a solid. The experiment consists of making it slowly roll on a plate with a small step, and following the contact dynamics after the drop reaches the bottom level at time *t* = 0 (see Movie 2 in the SI). The step height (0.5 mm) is significantly smaller than the drop diameter (2 mm) for minimizing the effect of impact (Fig. S2). We present in Fig. 4a the evolution of the contact radius *ℓ* for a marble filled with glycerol with either Ω =  3.4 μL (orange data) or Ω = 4.5 μL (blue data). The contact grows during ~3 ms, where it can be described by a scaling law with power 1/3 (dots), until it reaches the MP plateau *ℓ*_o_ = (2/3)^1/2^*R*^2^/*a* shown with dashes.

We can try to understand this unusual spreading law. The contact radius *ℓ* geometrically relates to the lowering δ of the center of mass of the drop, *ℓ* ^2^ ~ δ*R*, so that the vertical velocity of compression δ/*t* can be written *ℓ*^2^/*Rt*. If we balance the viscous force η(*ℓ*^2^/*Rt*)*ℓ* by the weight ρ*gR*^3^, we deduce the law of spreading for non-wetting rotating viscous drops, *ℓ*(*t*) ~ *R* (ρ*gRt*/η)^1/3^ whose exponent nicely describes the data in Fig. 4a. The model predicts a contact radius of 0.2 mm after 1 ms for a millimetric drop of glycerol, also in accord with the experiment. This gravitational spreading is original and observed for both pearls and marbles (see Fig. S3), with an exponent in time of 1/3 instead of 1/10 for wetting liquids^15^. For the parameters of our experiment, reaching a distance *ℓ*_o_ ≈ 400 µm in a non-wetting state (kinetics in *t*^1/3^, Fig. 4a) takes about 500 times longer than in a wetting situation (kinetics in *t*^1/10^). This “slow” dynamic eventually explains why the contact becomes velocity dependent above some threshold in *V*.

Since a rolling marble has a time *ℓ*/*V* to establish its contact, we can plug this time into the spreading law *ℓ*(*t*) and obtain the contact size, *ℓ* ~ *R* (ρ*gR*^2^/η*V*)^1/2^*ℓ*~ *ℓ*_o_ (γ/η*V*)^1/2^ ~ *ℓ*_o_ Ca^−1/2^. Hence, *ℓ* decreases with *V*, and it gets significantly smaller than the static MP contact *ℓ*_o_ ~ *R*^2^/*a* when the capillary number exceeds unity, opposite to the assumption Ca <1 in the MP model^4^. Contact shrinks (*ℓ* < *ℓ*_o_) if *V* is larger than γ/η, that is, a few centimeters per second for glycerol. Figures 2b and 3a confirm that both deviations from the MP law and contact retraction appear at a velocity of this magnitude. Injecting the dynamic contact *ℓ*(*V*) in the formula of the viscous torque (η*V*/*R*)*ℓ*^3^ yields (ρ*g*)^3/2^*R*^5^/(η*V*)^1/2^, which decreases with speed. Faster marbles have a smaller friction, thus an even faster speed, and so on: drops keep accelerating, which makes the descent non-stationary and leads to the “divergence” of the speed at some critical slope α_c_ (as seen in Fig. 2b), whose value is found by injecting a capillary number of order unity in the MP law. We find α_c_ ~ *R*/*a* and indeed observe in Fig. 4b that α_c_ increases linearly with the marble size. This implies that small marbles tend to be super-quick even at a small tilt—a paradox since viscous objects generally tend to be slower when smaller. Hence, the domain where the MP law is the most extraordinary (*R* < *a*) is also the one where its validity is the most limited, which explains a posteriori why experiments challenging this law were conducted at tilt angles as low as 4°^5^.

**Fig. 5:**
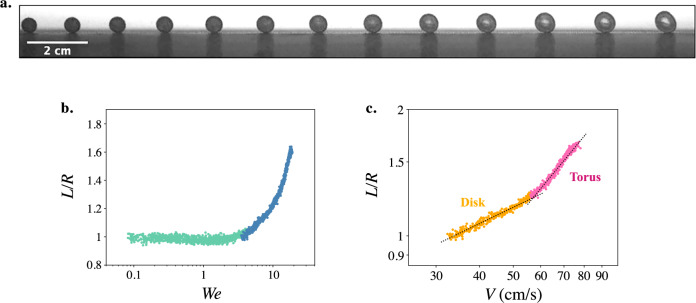
Centrifuged marbles. **a** Chronophotography of a marble of glycerol (*R* = 1.6 mm) as it runs down a plate tilted by α = 30° (camera tilted by the same angle, interframe time of 20 ms). The drop keeps on growing as its shape changes from discoidal to toroidal (white spot at the center). In the meantime, the speed increases from 55 to 85 cm/s, and it reaches its maximum when the size is maximum (Movie 3 in the SI). **b** Side radius *L* of a glycerol marble normalized by its initial radius *R* as a function of the number We = ρ*V*^2^*R*/γ that compares centrifugal force with surface tension. Marbles deform (*L* > *R*) if We exceeds a value of order unity. Colors correspond to experiments with α = 19° (green) and α = 30° (blue). **c** For the blue data, we plot the relative growth *L*/*R* as a function of the measured velocity *V* (logarithmic scales). We observe two successive regimes, corresponding to discoidal and toroidal drops, respectively, in orange and pink. Dots show slopes 2/5 and 1.

The existence of very fast regimes has other consequences: quickly rotating marbles can adopt exotic shapes arising from centrifugation. Aussillous reported that marbles on high slopes can look like peanuts or doughnuts^16^, two shapes predicted by Rayleigh, Poincaré, Chandrasekhar, and Scriven for freely revolving drops^17–20^. We expect to lose spherical shapes if the centrifugal force exceeds the capillary action, that is, if the Weber number We =  ρ*V*^2^*R*/γ (highly sensitive to *V*) is larger than some threshold of order unity. Figure 5a shows a marble on a plate inclined by 30°, viewed from the side by a camera tilted by the same angle. The photos capture both the instantaneous drop velocity and shape. At such tilts, a marble grows: centrifugation makes discoidal the initially spherical drop, with radius *L* larger than *R* by typically 30%; yet neither this number nor the speed are constant: the drop keeps on growing and accelerating, until it adopts the toroidal shape disclosed by a white spot at the disk center.

This qualitative description can be made more quantitative by measuring how the side radius *L* of a marble depends on its velocity *V*. Figure 5b confirms the role of centrifugation: the ratio *L*/*R* becomes larger than 1 if the Weber number exceeds a value of order unity—above which marbles quickly grow with We. For the sake of simplicity, we consider two asymptotic modes of deformation, a disk and a torus, with radius *L* and thickness *b* << *L*. On the one hand, assuming solid-like rotation, the balance of forces that fixes *L* can be written per unit volume ρ*V*^2^/*L* ∼ γ/*b*^2^, where the latter quantity is the gradient of Laplace pressure pointing to the center of the revolving liquid. On the other hand, volume conservation discriminates both shapes, being *R*^3^ ~ *L*^2^*b* for a disk and *R*^3^ ~ *Lb*^2^ for a torus. After eliminating *b*, we get the normalized size of both objects, that is *L*/*R* ~ (ρ*R*/γ)^1/5^ *V*^2/5^ for a disk, and *L*/*R* ~ (ρ*R*/γ)^1/2^ *V* for a torus^16^. Despite their asymptotic character, these laws drawn with dots in Fig. 5c quantitatively describe the data with prefactors 0.8 and 0.4. More qualitatively, we understand the fast-growing tendency in Fig. 5b: as marbles accelerate, their size becomes more and more sensitive to velocity.

Hence, the “acceleration” of the marbles is not driven by inertia but by their transformation, since viscosity acts in a region *ℓ* fixed by the marble shape (Fig. 3a). For a marble with a side radius *L* > *R*, we saw that *ℓ* scales as *R* (ρ*gRL*/η*V*)^1/2^. Injecting in this formula the distance *L*(*V*) for disks and tori, we get, respectively, *ℓ* ~ *R* (ρ^6^*g*^5^*R*^11^/γη^5^*V*^3^)^1/10^ and *ℓ* ~ *R* (ρ^3^*g*^2^*R*^5^/γη^2^)^1/4^, where the latter expression is independent of the velocity. Hence, we predict four consecutive regimes of contact, depending on the drop velocity. While *ℓ* tends to *ℓ*_o_ (MP limit) at low speed (regime I), we then expect a decrease of *ℓ* as *V*^−1/2^, when the rotating drop is still spherical but too quick to achieve a quasi-static contact (regime II); it is followed by a second, weaker decrease, as *V*^−3/10^, when the marble is discoidal (regime III); and the contact eventually reaches a minimum value for toroidal marbles, *ℓ* ~ *ℓ*_o_/Oh^1/2^, where the Ohnesorge number (Oh = η/(ργ*R*)^1/2^) is of order 5 for millimetric drops of glycerol (regime IV). These four regimes are shown with dotted lines in Fig. 3a, where they convincingly fit the data. The range of each regime is small (by definition), but their predicted magnitudes also agree with experiments: the fits in the figure are drawn with respective numerical coefficients of 1, 0.7, 0.7 and 0.6, all of order unity.

**Fig. 6:**
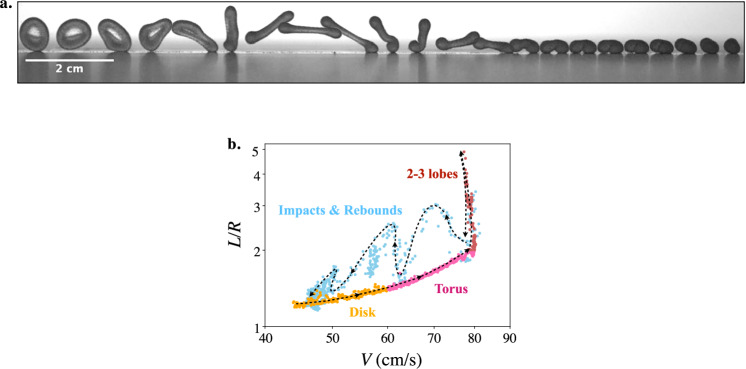
Acrobatics of viscous marbles. **a** Chronophotography of a marble of glycerol (*R* = 1.6 mm) running down a plate tilted by α = 30° (camera tilted by the same angle, images separated by 10 ms). When the toroidal marble becomes three-lobed, it takes off and spontaneously transforms into a two-lobed shape. Forced by gravity to land on the substrate, it slows down, reassembles and restarts a new cycle, explaining the wavy, periodic trajectory in Fig. 2a (Movie 4 in the SI). **b** Side size *L* of a glycerol marble normalized by its initial radius *R* = 1.6 mm on an incline (α = 30°) as a function of its speed *V*. *V* increases during the sequence of axisymmetric deformations (disc in orange, torus in pink); it remains roughly constant for flying 3- and 2-lobed shapes (in red) before decreasing with oscillations after successive impact and rebounds (in blue).

Viscous marbles on steep slopes gradually transform into tori, but Chandrasekhar, Scriven and Trinh argued that tori should be unstable^19–21^. We indeed observe their spontaneous transformation in a three-lobed shape (fourth image in Fig. 6a and Fig. S4), a configuration also reported for centimetric spinning water drops levitating in a strong magnet^22^. As seen in Fig. 6a, the loss of axisymmetry forces the liquid to take off, which makes it adopt the two-lobed shape predicted by Chandrasekhar to be more stable^19^. This “peanut” flies in the air for typically 30 ms before crashing on the ground, where it dissipates kinetic energy, an effect favored by the increase of solid/liquid area at landing. It can rebound and crash again, and the successive impacts also dampen rotation so that marbles recover a roughly spherical or discoidal shape, from which they restart their acrobatics, explaining the wavy, periodic trajectory in Fig. 2a—a cycle of transformations seen in Fig. S5 to be unsensitive to changes in the size and density of the coating grains. The instantaneous speed decreases between ∼80 cm/s (for the torus before its instability) and ∼45 cm/s (after impact), which sets its average value in Fig. 2b. Figure S6 reveals how the speed varies within a cycle of transformation, with successive increase, decrease and oscillations. Figure S7 shows that the cycle is independent of the viscosity, a property tested between 800 and 1400 mPa.s.

This complex sequence is further illustrated in Fig. 6b, where we plot the relative growth *L*/*R* of the object as a function of its velocity *V* during one cycle of transformations. The initial disk (in orange) accelerates as it reduces its contact with the substrate, until it becomes a torus (in pink) that keeps on accelerating and growing. This induces its transition to the three- and two-lobed shapes (in red) that roughly keep their velocity in air, owing to inertia. Then, impacts (in blue) strongly decrease the marble velocity and size, with oscillations due to successive rebounds, which eventually brings the system back to its initial state.

In conclusion, viscous marbles on weakly inclined solids are quick (MP model), but speed increases by one order of magnitude when solids are tilted by “daily-life angles”, that of windshields or roofs, for instance, which might define the domain of applications where non-wetting reaches its most spectacular limit, in terms of mobility. This ultra-quick regime arises from the reduction of the contact when marbles become fast enough, and then even faster, due to this reduction. Because viscous drops are also rotating, a cascade of transformations is observed, which, together with the reduction of contact, makes the dynamics non-stationary.

The reason for these fast regimes is paradoxical. While viscosity generally limits the speed of liquids, it provides here the opposite effect: drops are ultra-quick because a viscous liquid needs time to establish contact with its substrate. In addition, speed amplifies the effect, owing to the rotation-induced transformations of the marbles. This rotation also contributes to avoid the fragmentation of the liquid, since it localizes viscous dissipation in a region of size *ℓ* much smaller than *R*, the typical size of the contact for a “regular” drop. Contrasting with fast regular drops, viscous marbles thus do not elongate, which prevents the fragmentation of the liquid: despite a high velocity, marbles remain whole along their run—a property of obvious practical interest.

As natural extensions of this work, we can think of varying either the nature of the liquid that constitutes the marbles, or their coating. (1) The case of intermediate viscosities should be rich and complex, since it can add inertial effects to the picture, but also jeopardize the condition for generating rotation, a key fact in this paper. Another situation of practical interest concerns non-Newtonian fluids, where dynamics should be multifaceted, since it then depends on rheology. This should lead a priori to all kinds of velocity regimes, according to the nature of the liquid. 2) We reported that more dilute coatings or smaller grains generate the same results (Fig. S5), but we expect differences if we concentrate the grains at the surface to the point of jamming—which induces another source of dissipation. Conversely, it would be worth looking at the dynamics of non-coated, viscous, poorly wetting “pearls”. Then, we may slightly relax the constraint of non-wetting—so that we anticipate transitions between the regimes reported here and more classical dynamics. In brief, all these studies would classify at large the high mobility of non-wetting objects, that is, their key property for applications^6,7^.

## Methods

### Marble preparation

Liquid marbles were formed by rolling liquid drops in a micrometric hydrophobic powder (Lycopodium spores, Fluka), yielding a particle monolayer with a surface coverage of ~80%. Lower surface coverages were achieved by post-formation dilution from smaller droplets.

### Image-based measurements

Measurements were obtained from side-view recordings of marbles moving down a 2.5-m-long incline made of steel. Videos were acquired using a high-speed camera (Phantom) at frame rates ranging from 300 to 8000 fps, with variable magnification. The slope angle was measured using a digital inclinometer (RS Pro; accuracy ±0.2°). The radii of the contact made by marbles with their substrate were measured manually from individual frames. The marbles' position and shape were extracted using automated image analysis, and the velocity was determined by numerical differentiation (see SI).

## Supplementary information

Supplementary Information

Description of Additional Supplementary Files

Supplementary Movie 1a

Supplementary Movie 1b

Supplementary Movie 2

Supplementary Movie 3

Supplementary Movie 4

Transparent Peer Review file

## Data Availability

Examples of videos are provided in the Supplementary Materials (Videos 1–4). A folder containing all the data displayed in the figures is accessible at 10.5281/zenodo.18197695. Additional information is available from the corresponding author upon request.

## Code availability

The code used in the current study is shown in the Supplementary Materials.
