# Supplementary material for: Ultra-quick dynamics and acrobatics of viscous marbles
Source: Nat Commun. 2026 May 13;17:6357. doi: 10.1038/s41467-026-69128-2 (PMC13376779; doi:10.1038/s41467-026-69128-2)
Supplement: Supplementary file 1 — Supplementary Information [file 41467_2026_69128_MOESM1_ESM.pdf]

Ultra-quick dynamics and acrobatics of viscous marbles

**Auriane Huyghues Despointes[1], Yui Takai[1,2],
Shoko Ii[1], Timothée Mouterde[2] & David Quéré[1,*]**

*1. Physique et Mécanique des Milieux Hétérogènes, UMR 7636 du CNRS,
PSL Research University, ESPCI-Paris, France.*

*2. Department of Mechanical Engineering, School of Engineering,
The University of Tokyo, Tokyo, Japan.*

* Corresponding author (david.quere@espci.fr)

Supplementary materials: Additional experiments & information

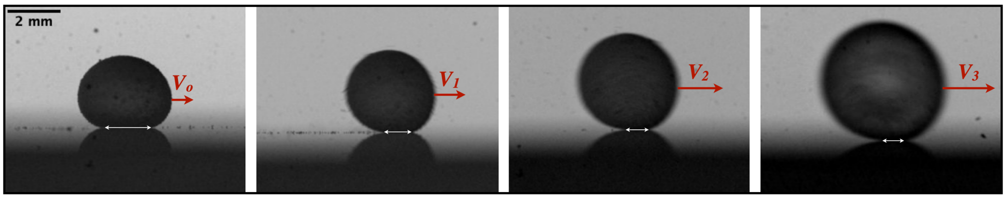

Figure S1. Dynamic contact. Measurement of the size of the dynamic contact of a glycerol marble (Ω = 18 μL, instantaneous speed V) rolling down an incline (camera tilted by the same angle α). From left to right: α_o = 9° and V_o = 2.5 cm/s, α_1 = 19° and V_1 = 13 cm/s, α_2 = 22° and V_2 = 43 cm/s, α_3 = 24° and V_3 = 65 cm/s. As the speed increases, the drop grows owing to centrifugation and the contact size (marked by arrows) decreases, from 1.75 mm in the first case to 0.75 mm in the last one.

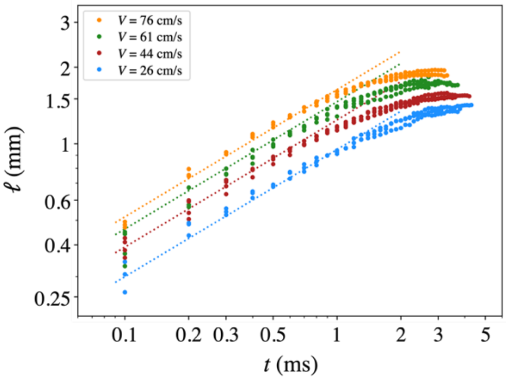

Figure S2. Impact of a marble. Time evolution of the contact radius ℓ of a glycerol marble (R = 1.6 mm) that impacts a flat solid at a velocity V. The contact size is geometrically related to the lowering δ of the center of gravity of the drop as it impacts its substrate $\ell \sim (R\delta)^{1/2}$, which, after introducing the impact velocity, $\delta \sim Vt$, yields $\ell \sim (RVt)^{1/2}$. The spreading exponent of 1/2 indeed fits the data at short time, as shown by the dots, where a common numerical factor of 1.5 has been introduced. This dynamic is different from that of a spreading drop (V = 0) shown to scale as $t^{1/3}$ in the figure 4a of the accompanying paper and in figure S3.

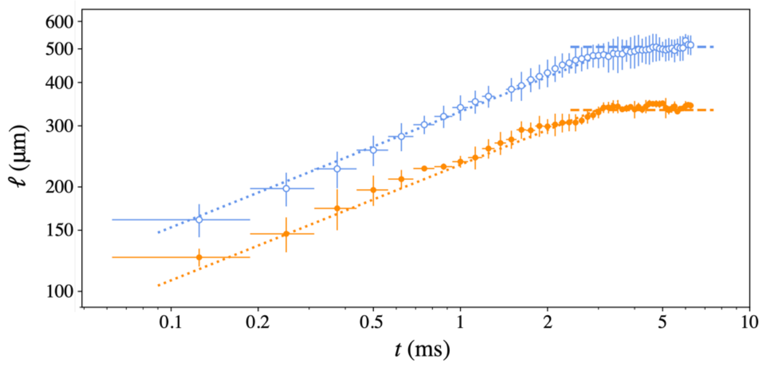

Figure S3. Gravitational spreading of a viscous pearl. Time evolution of the contact radius $\ell(t)$ for a rolling glycerol drop (Ω = 7.5 µL, blue data) contacting at t = 0 a super-hydrophobic solid (Glaco-coated glass). The distance ℓ (open symbols) increases as $t^{1/3}$ before reaching its static value ℓ_o. It is compared to the contact of a glycerol marble (Ω = 3.4 µL, orange data taken from figure 4a). Dots show our model with an identical numerical prefactor of 1.1, and the shift between the two experiments just arises from the difference in drop volume.

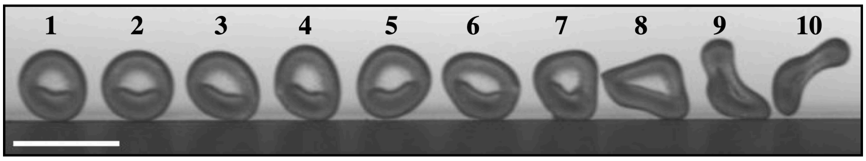

Figure S4. Trilobe. Chronophotograph of a glycerol marble with R = 1.9 mm running down a plate tilted by 30° (camera tilted by the same angle, images separated by 10 ms, scalebar 1 cm). The torus (images 1 and 2) spontaneously transforms (images 3-6) into a trilobe visible (images 7-9). This shape forces its take-off, which irreversibly transforms it into a two-lobed shape (image 10). The drop is larger than that in figure 6a, which provides a better view of the trilobe.

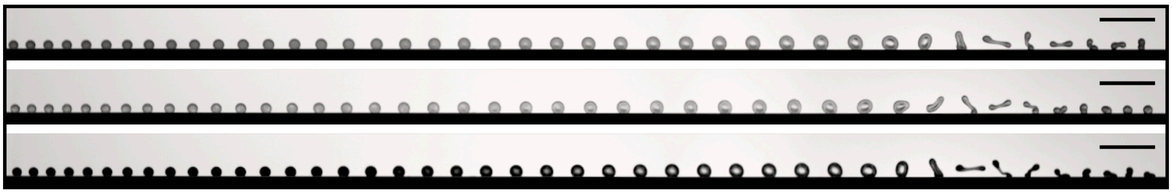

Figure S5. Influence of the grains on the acrobatics of marbles. Chronophotographs of glycerol marbles (Ω = 18 μL) running down a plate tilted by 25° (camera tilted by the same angle, interframe time 17 ms, scalebar 2 cm). From top to bottom: 1) Marble covered by lycopodium grains with a surface packing of 0.8 (measured under a microscope). 2) Marble made of the same grains with a packing of 0.5, obtained by diluting a smaller marble until it reaches the volume Ω. 3) Marble made of iron filings with a diameter of 4 µm instead of 30 µm for lycopodium. The hydrophilic iron filings are made hydrophobic by treating them with hydrophobic nanobeads (purchased from Glaco). The drop dynamics are identical for the three systems, showing the generality of our findings, found to be independent of the coverage density and size.

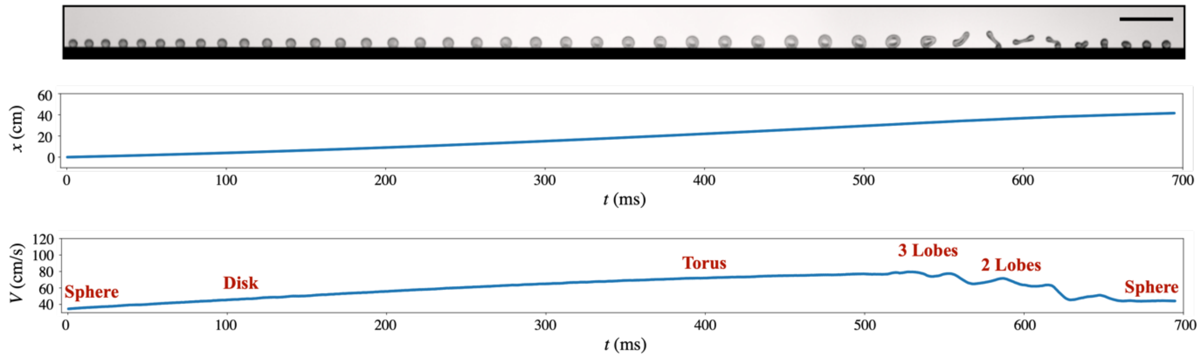

Figure S6. Instantaneous velocity of an acrobatic marble. If we extract from the marble trajectory $x(t)$ (figure 2a in the accompanying paper) its velocity V in the regime of acrobatics (here, $\alpha = 25°$), we can follow how V varies with time in a cycle of deformation, and correlate the velocity with the shape – indicated in the figure and seen in the chronophotograph at the top (scalebar 2 cm, interframe time 17 ms). In this experiment, the marble is made of glycerol with $\Omega = 18$ µL. Its velocity increases due to the contact reduction, see figure 3a, until a trilobe forms and takes off, transforms in a bilobe that lands and bounces – a sequence that produces a slowing down (with oscillations) due to the loss of energy in the successive shocks with the solid.

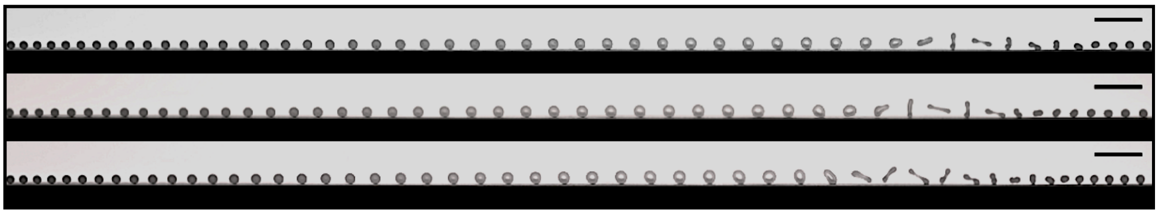

Figure S7. Influence of the viscosity on the acrobatics of marbles. Chronophotographs of water-glycerol marble ($\Omega = 18$ µL) running down a plate tilted by 25° (camera tilted by the same angle; interframe time 17 ms; scalebar 2 cm). From top to bottom, the marble viscosity η is 800, 1100 and 1400 mPa.s. The dynamics hardly depend on η: the sequence of deformations, the velocities associated with them and the duration of the cycle are comparable. However, take-off occurs slightly earlier when the viscosity is higher, in agreement with our arguments in the accompanying paper: the dynamic contact being smaller when the viscosity is higher, drops are quicker, which triggers the shape transition earlier.

Trajectory and velocity extraction from the movies (python code)

```
# -*- coding: utf-8 -*-
# Supplementary script — droplet centroid tracking and kinematics on a pre-binarized video.
# Assumptions (experiment-side): input .avi is strictly binary 0/255 (droplet=white, background=black),
# single connected droplet per analyzed frame, known frame rate (pps) and spatial calibration (ppm).
# The script reads frames, extracts the largest white component, measures its centroid and a proxy size
# via the minimum-area rectangle, builds a time series, and computes distance, speed, and acceleration.

import cv2
import numpy as np
import pandas as pd
import matplotlib.pyplot as plt
from scipy.signal import savgol_filter


def insert_speed(df: pd.DataFrame, window: int = 11, polyorder: int = 2) -> None:
    """
    Compute velocity and acceleration using Savitzky-Golay differentiation.
    Columns added / overwritten:
        - 'speed (m/s)'
        - 'a (m/s^2)'
    """
    T = df['time (s)'].to_numpy()
    d = df['distance (m)'].to_numpy()
    dt = np.mean(np.diff(T))      # mean timestep (robust to a few dropped/duplicated frames)

    # First and second derivatives of distance(t); delta=dt sets the physical time scale.
    v = savgol_filter(d, window_length=window, polyorder=polyorder,
                      deriv=1, delta=dt)
    a = savgol_filter(d, window_length=window, polyorder=polyorder,
                      deriv=2, delta=dt)

    df["speed (m/s)"] = v
    df["a (m/s^2)"] = a


# ----------------------------------------------------------------
# PARAMETERS
# ----------------------------------------------------------------
path = ('')         # Base path to the video (without extension). Expects '<path>.avi'.
pps = 3000          # Frames per second (pictures per second) from acquisition.
ppm = 3.0364        # Pixels per millimeter (global spatial calibration).
mpp = 1 / ppm       # Millimeters per pixel (convenience scalar).
N   = 51            # Savitzky-Golay window length (odd; N > polyorder).
# ----------------------------------------------------------------
# LOAD VIDEO
# ----------------------------------------------------------------
# Input MUST already be binary (0/255): droplet in white on black background.
cap = cv2.VideoCapture(path + '.avi')
frame_total = int(cap.get(cv2.CAP_PROP_FRAME_COUNT))
print(f"Total Frames: {frame_total}")

# Time series containers
T, Xc, Yc, Max_L, Max_W = [], [], [], [], []

while True:
    ret, frame = cap.read()
    if not ret:
        break  # end of stream

    # Convert frame index to physical time using the acquisition frame rate.
    index = int(cap.get(cv2.CAP_PROP_POS_FRAMES))
    t = index / pps

    # Normalize to grayscale; handles both single-channel and BGR inputs uniformly.
    gray = cv2.cvtColor(frame, cv2.COLOR_BGR2GRAY)

    # Safety re-thresholding; for strictly binary input this preserves {0,255}.
    thresh = cv2.threshold(gray, 100, 255, cv2.THRESH_BINARY)[1]

    # Extract external contours; the droplet is assumed to be the largest white component.
    contours, _ = cv2.findContours(thresh, cv2.RETR_EXTERNAL, cv2.CHAIN_APPROX_SIMPLE

    # Fit a minimum-area rectangle to the largest component.
    # OpenCV returns: center (xc,yc), side lengths (w,h) in pixels, and a rotation angle.
    rect = cv2.minAreaRect(max(contours, key=cv2.contourArea))
    (xc, yc), (w, h), _ = rect

    # Define footprint proxies: major/minor sides in millimeters (no ellipse fit).
    max_l = max(w, h) * mpp
    max_w = min(w, h) * mpp

    # Record time, centroid (converted to mm), and size proxies.
    T.append(t)
    Xc.append(xc * mpp)
    Yc.append(yc * mpp)
    Max_L.append(max_l)
    Max_W.append(max_w)

cap.release()
```

```
# ----------------------------------------------------------------
# DATAFRAME + DERIVED QUANTITIES
# ----------------------------------------------------------------
E = pd.DataFrame({
    'time (s)': T,
    'xc (mm)': Xc,
    'yc (mm)': Yc,
    'Max Length (mm)': Max_L,
    'Max Width (mm)': Max_W,
})

# In-plane displacement from the first detected position (planar motion assumed, no perspective correction).
dx = E['xc (mm)'] - E['xc (mm)'].iloc[0]
dy = E['yc (mm)'] - E['yc (mm)'].iloc[0]
E['distance (m)'] = np.sqrt(dx**2 + dy**2) * 1e-3   # mm → m

# Speed and acceleration from distance(t) via Savitzky–Golay differentiation.
insert_speed(E, N)

# Mean radius proxy (mm): r = (L + W) / 4; for a circle with L≈W≈2R, r≈R.
E['r (mm)'] = (E['Max Length (mm)'] + E['Max Width (mm)']) / 4

# Persist full dataset (avoids CSV formatting issues; preserves dtypes/precision).
E.to_pickle(path + ".pkl")

# ----------------------------------------------------------------
# PLOTS
# ----------------------------------------------------------------
# Quick diagnostics: distance and speed vs time, expressed in cm and cm/s for readability.
fig1, ax1 = plt.subplots()
ax1.plot(E['time (s)'], E['distance (m)'] * 100, '.')
ax1.set_xlabel('$t$ (s)')
ax1.set_ylabel('$d$ (cm)')

fig2, ax2 = plt.subplots()
ax2.plot(E['time (s)'], E['speed (m/s)'] * 100, '.')
ax2.set_xlabel('$t$ (s)')
ax2.set_ylabel('$v$ (cm/s)')
```
