# Supplementary material for: Ultra-quick dynamics and acrobatics of viscous marbles
Source: Nat Commun. 2026 May 13;17:6357. doi: 10.1038/s41467-026-69128-2 (PMC13376779; doi:10.1038/s41467-026-69128-2)
Supplement: Supplementary file 2 — Description of Additional Supplementary Files [file 41467_2026_69128_MOESM2_ESM.pdf]

Ultra-quick dynamics and acrobatics of viscous marbles

Auriane Huyghues Despointes[1], Yui Takai[1,2],
Shoko Ii[1], Timothée Mouterde[2] & David Quéré[1,*]

1. Physique et Mécanique des Milieux Hétérogènes, UMR 7636 du CNRS,
PSL Research University, ESPCI-Paris, France.

2. Department of Mechanical Engineering, School of Engineering,
The University of Tokyo, Tokyo, Japan.

* Corresponding author (david.quere@espci.fr)

Additional Supplementary Files

We provide below the legends of the supplementary movies.

File Name: Supplementary Movie 1

Movie Title: Ultra-quick dynamics of viscous marbles.

Description: **a.** Video of a marble of glycerol (Ω = 18 μL) as it runs down a plate tilted (camera tilted by the same angle, scalebar corresponding to 2 cm) by α = 12° (top movie) or α = 35° (bottom movie). While the marble is stationary in the first case (Mahadevan-Pomeau regime), its velocity and shape oscillate in the second case. Movies shown in real time. **b.** Same movies as previously, yet slowed down 5 times.

File Name: Supplementary Movie 2

Movie Title: Spreading of a marble.

Description: Video of a marble of glycerol (volume of 4 μL) as it contacts a flat solid (video slowed down 200 times). After 3 ms, the contact stops growing and reaches its static value.

File Name: Supplementary Movie 3

Movie Title: Centrifuged marbles.

Description: Video of a marble of glycerol (volume of 18 μL) running down a plate tilted by 30° (camera tilted by the same angle, video slowed down 50 times). The drop keeps on growing (diameter from 4 mm to 6 mm) and accelerating (from 55 cm/s to 85 cm/s), which makes it become toroidal.

File Name: Supplementary Movie 4

Movie Title: Acrobatics of viscous marbles.

Description: Video of a marble of glycerol (volume of 18 μL) running down a plate tilted by 30° (camera tilted by the same angle, the video slowed down 50 times). This movie follows that in video 3. The toroidal marble is unstable and it adopts a three-lobed shape that spontaneously takes off and transforms into a two-lobed shape. Forced by gravity to land on the substrate, it slows down (from 80 cm/s to 40 cm/s) and reassembles in a globular object before restarting a new cycle.
