# Supplementary material for: Ultra-quick dynamics and acrobatics of viscous marbles
Source: Nat Commun. 2026 May 13;17:6357. doi: 10.1038/s41467-026-69128-2 (PMC13376779; doi:10.1038/s41467-026-69128-2)
Supplement: Supplementary file 8 — Transparent Peer Review file [file 41467_2026_69128_MOESM8_ESM.pdf]

nature portfolio

Peer Review File

Ultra-quick dynamics and acrobatics of viscous marbles

Corresponding Author: Professor David Quéré

This file contains all reviewer reports in order by version, followed by all author rebuttals in order by version.

Version 0:

Reviewer comments:

Reviewer #1

(Remarks to the Author)
The authors report an intriguing and counterintuitive phenomenon: unusually high-speed motion in non-wetting viscous drops. They convincingly connect this behavior to the interplay between viscosity, rotation, and dynamic contact reduction. The results are well motivated and potentially impactful, both fundamentally and for applications involving liquid-repellent materials. The manuscript is well written and clearly organized. I recommend publication after the following questions and comments are addressed.

1. While non-wetting behavior is often associated with micro/nano-structured surfaces (e.g., superoleophobic surfaces), viscous oil drops can also exhibit non-wetting characteristics. Since liquid marbles are an archetype of non-wetting drops, it would be helpful to clarify whether the results presented here are expected to hold for viscous drops on superoleophobic surfaces as well. In other words, are liquid marbles a special case? This seems particularly relevant given that the authors note a gravitational spreading law for viscous marbles with a time exponent completely different from that of wetting liquids or impacting drops.

2. In line 57, the speed should be defined explicitly as the symbol V for consistency.

3. In Fig. 1, the label for the contact radius should be corrected to '2 ℓ_0'.

4. In the caption of Fig. 2a, the authors state that the descent is stationary at low angles ($\alpha < 18°$) but becomes wavy and much quicker when $\alpha > 20°$. If I understand correctly, the latter case corresponds to 'non-stationary' dynamics. It would be helpful to define this term explicitly here, as this term can also related to the initial acceleration stage and is not clearly defined elsewhere in the manuscript.

5. In Fig. 3a, the y-axis label should be corrected to 'ℓ/ℓ_0'.

6. In Fig. 3b, there appears to be no regime with a slope of –3, as observed in Fig. 3a. Since Fig. 3b includes both stationary and non-stationary cases, could the authors clarify why the stationary regime does not appear? Does this imply that $\alpha = 9°$ already exceeds the critical angle α_c?

7. During the 'acrobatics' of viscous marbles, could the redistribution of surface grains influence the observed dynamics? For example, by creating local jamming that affects the marble's shape or mobility? It would be interesting to discuss whether such granular rearrangements contribute to the motion/morphing, beyond the purely viscous effects of the liquid itself.

Reviewer #2

(Remarks to the Author)
The manuscript entitled "Acrobatics of Viscous Marbles" presents a rigorous examination of the dynamics of non-wetting viscous droplets on inclined surfaces, extending the Mahadevan-Pomeau model to regimes characterized by elevated driving forces and revealing a critical threshold at which velocities escalate by an order of magnitude—attaining values up to 50 cm/s, commensurate with those of non-viscous aqueous droplets despite a 1000-fold viscosity differential—attributable to dynamic diminution of substrate contact and rotational shape instabilities paradoxically engendered by viscosity. This study contributes substantially to soft matter physics by elucidating viscosity's counterintuitive facilitative role in retarding contact equilibration, thereby attenuating substrate friction in high-velocity regimes; delineating sequential morphological transitions

from spherical to discoidal, toroidal, trilobate, and bilobate configurations, supported by a novel $t^{1/3}$ gravitational spreading law distinctive to non-wetting viscous systems; and generalizing the Mahadevan-Pomeau framework to incorporate four contact regimes that account for non-stationary dynamics via scalable theoretical constructs applicable to varied fluids and substrates. Bolstered by meticulous experiments employing high-speed imaging and scaling analyses, the investigation demonstrates notable originality and interdisciplinary significance, with implications for microfluidics, self-cleaning materials, and viscous fluid transport, rendering it highly congruent with Nature Communications' emphasis on innovative, broadly accessible scholarship. Although the exposition is robust, refinements in mathematical derivations, quantitative specificity, and graphical elements would enhance reproducibility and lucidity, thereby strengthening its prospects for publication.

1. The title is engaging and evocative, suitable for a multidisciplinary journal; however, consider making it more descriptive to emphasize the core discovery, such as "Ultra-quick dynamics and shape instabilities in rolling viscous liquid marbles on inclines." The abstract effectively summarizes the key results and paradoxical role of viscosity but could better highlight broader implications, such as relevance to anti-fouling technologies or droplet-based robotics. Rephrase the final sentence for clarity: "Paradoxically, this super-quick regime results from viscosity, since this quantity is responsible for the contact dynamics: viscous drops slowly relax to equilibrium, so that fast ones have not enough time to establish an equilibrium contact, which makes them faster" to "Paradoxically, viscosity enables this regime by impeding rapid contact equilibration, thereby reducing substrate interactions in fast-moving drops."
2. The introduction adeptly contextualizes non-wetting droplets. It references seminal works, but it could be strengthened by explicitly addressing the limitations of prior studies (e.g., confinement to low tilt angles) and delineating how this research expands upon them. Incorporate a brief discussion of interdisciplinary relevance, such as connections to materials science or biomechanics, to broaden appeal for Nature Communications readers.
3. The description of marble preparation, viscosity measurements, and imaging techniques is comprehensive, facilitating reproducibility. To enhance rigor, provide additional specifics: the supplier and exact properties of lycopodium spores, surface roughness of the steel strips (e.g., root-mean-square value), and calibration methods for tilt angles. Include error propagation analyses for velocity extractions and consider sharing raw data or analysis scripts in supplementary materials.
4. Figure 1: The panels clearly illustrate the marble's static and dynamic states. Improve the caption by adding quantitative parameters (e.g., "Drop volume $\Omega = 5$ µL, viscosity $\eta = 1000$ mPa·s"). Ensure scale bars are present in both subfigures and label the contact radius λ_o directly on the image. Increase image resolution to prevent pixelation in the final submission.
5. Figure 2: The trajectories in panel (a) effectively contrast stationary and oscillatory motions, while panel (b) highlights deviations from the Mahadevan-Pomeau model. Add error bars to velocities in (b) to quantify measurement uncertainty. Specify the fitted equation for the dotted Mahadevan-Pomeau line in the caption or methods. Integrate references to supplementary Movie 1 more prominently, ensuring movies include annotations like timestamps and scale indicators.
6. Figure 3: The normalization and scaling analyses in panels (a) and (b) robustly support the dynamic contact reduction hypothesis. Replace the color gradient (warm to cold for angles) with a discrete legend for improved accessibility. Justify numerical coefficients in scaling fits (e.g., 0.9, 0.7) more explicitly in the text. Add a schematic inset depicting the four contact regimes to aid comprehension.
7. Figure 4: The derived $t^{1/3}$ scaling for gravitational spreading is innovative; provide a quantitative comparison to wetting drop literature (e.g., reference [12]). In panel (b), bolster the linear fit for α_c versus R/a with additional data points at smaller R values. Standardize axis labels (e.g., consistently use "R/a") and include units throughout.
8. Figures 5 & 6: Those time-lapse shots really bring the "acrobatics" to life—key to the story. Jazz them up with velocity arrows or outlines on each frame. In Figure 5c, mark the regimes (like disk, torus) right on the plot. For Figure 6b, include a simple cycle diagram to clarify the transitions. Ensure the supplements (S1–S3 and Movies 3–4) are consistent, maintaining the same slowdown rates and scale bars.
9. The Mahadevan-Pomeau model extensions and scaling arguments are logical, but the l(V) derivation should be further developed using lubrication theory or dimensional analysis. Note limitations, such as powder coating effects on dissipation; recommend testing different grain sizes. Calculate Reynolds numbers for all velocities to confirm the low-Re assumption.
10. The paradoxical viscosity effect is well explained, but its practical uses—such as improving the movement of viscous droplets in pharmaceutical delivery—should be expanded on. It's also essential to highlight future research directions, such as investigating intermediate viscosities, exploring non-Newtonian fluids, or studying uncoated pearls. Additionally, references should be updated to include recent studies on liquid marbles published after 2020 to ensure the discussion is comprehensive.
11. The tenfold increase in velocity and related instabilities present original findings that challenge current paradigms with measurable data. A clearer comparison to other non-wetting systems and supporting data for the "tenfold" claim would strengthen the work. Exploring scalability and practical applications could further enhance its real-world relevance.

Reviewer #3

(Remarks to the Author)
The paper presents very interesting results, and I strongly recommend it for publication.
Remarks.
1. The paper reports the viscosity-driven phenomena. Perhaps, the explicit calculation of the Reynolds and capillary numbers will be useful for a reader.
2. The viscosity is strongly dependent on temperature; thus, the experiments carried out under different temperatures are strongly recommended.
3. Does the reported effect depend on the coating of the viscous marbles?
4. Perhaps, the paper:
On the Nature of the Friction between Nonstick Droplets and Solid Substrates, Langmuir 2010, 26, 15, 12479–12482, will be useful for the authors.

Version 1:

Reviewer comments:

Reviewer #1

(Remarks to the Author)
The authors have fully addressed all of my concerns. I recommend the manuscript for publication.

Reviewer #2

(Remarks to the Author)
Accept as is.

Reviewer #3

(Remarks to the Author)
The authors carefully addressed the remarks suggested by the reviewers. The paper is publishable.

Open Access This Peer Review File is licensed under a Creative Commons Attribution 4.0 International License, which permits use, sharing, adaptation, distribution and reproduction in any medium or format, as long as you give appropriate credit to the original author(s) and the source, provide a link to the Creative Commons license, and indicate if changes were made.
In cases where reviewers are anonymous, credit should be given to 'Anonymous Referee' and the source.
The images or other third party material in this Peer Review File are included in the article's Creative Commons license, unless indicated otherwise in a credit line to the material. If material is not included in the article's Creative Commons license and your intended use is not permitted by statutory regulation or exceeds the permitted use, you will need to obtain permission directly from the copyright holder.
To view a copy of this license, visit https://creativecommons.org/licenses/by/4.0/

We would like to thank the reviewers for carefully reading the paper. We are glad they all found the paper worth being published in *Nature Communications*. We elaborated below our response to each of their specific enquiries and marked in red our revisions in the manuscript. We also took this opportunity to read again the whole paper and make a few stylistic corrections (in red, as well).

Referee 1

1. While non-wetting behavior is often associated with micro/nano-structured surfaces (e.g., superoleophobic surfaces), viscous oil drops can also exhibit non-wetting characteristics. Since liquid marbles are an archetype of non-wetting drops, it would be helpful to clarify whether the results presented here are expected to hold for viscous drops on superoleophobic surfaces as well. In other words, are liquid marbles a special case? This seems particularly relevant given that the authors note a gravitational spreading law for viscous marbles with a time exponent completely different from that of wetting liquids or impacting drops.

The phenomena observed in our paper rely on two conditions: droplets must be both non-wetting and viscous to ensure solid-like rotation. To the best of our knowledge, the only system exhibiting *total* non-wetting is liquid marbles (*Soft Matter* **21**, 4807, 2025). As noted by the referee, oil on super-oleophobic surfaces can have high contact angles θ (for instance, 156° for hexadecane on soot, in *Science* **335,** 67, 2012), yet not as large than aqueous solutions nor marbles. As a consequence, the contact size is then generally given by geometry ($\ell \approx R(\pi-\theta)$) so that drops cannot follow anymore the MP law. They are slower and thus not likely to exhibit the regimes of high speed described in our study.

This is for oil, the case mentioned in the referee's report. For aqueous solutions on super-hydrophobic materials, the situation is different. These liquid pearls can have contact angles of 170°, a value now large enough to induce fast dynamics, with shape transitions and related dynamics. We added a note at the end of the paper to emphasize the relevance of a future research on this topic. We also provide a new figure in the SI (figure S3) where we show that viscous pearls follow the same gravitational spreading dynamics as marbles (in phase with the final sentence of the referee in the above paragraph).

2. In line 57, the speed should be defined explicitly as the symbol V for consistency.

Thank you for pointing out this lack of precision; we added the symbol in the revised manuscript.

3. In Fig. 1, the label for the contact radius should be corrected to '$2\ell_0$'.

Thank you for pointing out this error, which we corrected.

4. In the caption of Fig. 2a, the authors state that the descent is stationary at low angles (α < 18°) but becomes wavy and much quicker when α > 20°. If I understand correctly, the latter case corresponds to 'non-stationary' dynamics. It would be helpful to define this term explicitly here, as this term can also relate to the initial acceleration stage and is not clearly defined elsewhere in the manuscript.

Thank you for pointing out this lack of clarity; we have modified the caption accordingly and now define the words "stationary" and "non-stationary". Since the implicit question about the initial stage of acceleration is of interest, we also added a few lines about it at the end of the first paragraph in page 4.

5. In Fig. 3a, the y-axis label should be corrected to 'ℓ/ℓ_0'.

Thank you for pointing out this error; we corrected it.

6. In Fig. 3b, there appears to be no regime with a slope of −3, as observed in Fig. 3a. Since Fig. 3b includes both stationary and non-stationary cases, could the authors clarify why the stationary regime does not appear? Does this imply that α = 9° already exceeds the critical angle α_c?

Thank you for this question, which reveals a lack of clarity in this section. The confusion comes partially from the fact that there was an error in the x-axis caption of figure 3b, where it was written V/V_o while it was just V. Hence, we cannot do a direct comparison with the juxtaposed figure 3a. We now corrected, and switched the two figures (see further).

Confusion could also arise from the subtlety of the physics at stake, which we clarified. More specifically, we improved the description of figure 2b in our text, switched figures 3a and 3b, since figure 3a defines what comes next in the paper. To make it clear, we now introduce four zones (I to IV) in the new figure 3a, corresponding to the different regimes of contact, when this contact is dynamical. As specified in the revised caption, Zone I is the only one where a stationary regime of descent is observed, which we describe more precisely and further discuss in the new figure 3b. The angle of 9° belongs to this regime and it is thus lower than α_c, which, we think, is clear in the revised figures.

7. During the 'acrobatics' of viscous marbles, could the redistribution of surface grains influence the observed dynamics? For example, by creating local jamming that affects the marble's shape or mobility? It would be interesting to discuss whether such granular rearrangements contribute to the motion/morphing, beyond the purely viscous effects of the liquid itself.

This is a stimulating question. We do not think that the grains influence the acrobatic dynamics. To support this point, we made new experiments and added a figure in the SI (figure S5), which compares the acrobatics and trajectories of three different marbles: a “classical” one (surface grain density of 0.8), a marble with less grains on the surface (density of 0.5), and a marble made with much smaller grains (4 µm instead of 30 µm). We observe that dynamics are identical for all three objects. As stated by the referee, jamming might occur on marbles, but it only does at higher concentrations of grains – which must be made “on purpose” by some specific method, so that it does not represent a typical case.

Referee 2

1. The title is engaging and evocative, suitable for a multidisciplinary journal; however, consider making it more descriptive to emphasize the core discovery, such as "Ultra-quick dynamics and shape instabilities in rolling viscous liquid marbles on inclines." The abstract effectively summarizes the key results and paradoxical role of viscosity but could better highlight broader implications, such as relevance to anti-fouling technologies or droplet-based robotics. Rephrase the final sentence for clarity: "Paradoxically, this super-quick regime results from viscosity, since this quantity is responsible for the contact dynamics: viscous drops slowly relax to equilibrium, so that fast ones have not enough time to establish an equilibrium contact, which makes them faster" to "Paradoxically, viscosity enables this regime by impeding rapid contact equilibration, thereby reducing substrate interactions in fast-moving drops."

Thank you for these different suggestions. 1) We understand the point about the title, even if our usual style privileges short titles. We found here a compromise between our previous title and the one suggested by the referee, which makes us happy, in particular because it highlights one of our main findings – that is regimes of high velocity despite high viscosity. 2) This is one unique case where we would prefer not to follow the referee’s advice, the reason being that we do not wish to include general sentences about applications in an abstract. 3) Thanks a lot for improving so obviously the final sentence of our abstract: it was a pleasure to adopt the new sentence proposed by the referee.

2. The introduction adeptly contextualizes non-wetting droplets. It references seminal works, but it could be strengthened by explicitly addressing the limitations of prior studies (e.g., confinement to low tilt angles) and delineating how this research expands upon them. Incorporate a brief discussion of interdisciplinary

relevance, such as connections to materials science or biomechanics, to broaden appeal for Nature Communications readers.

Our introduction was indeed too quick. Following the referee's suggestion, we expanded it (on page 2), quote new recent references, and briefly introduce what will be the originality/purpose of our research.

3. The description of marble preparation, viscosity measurements, and imaging techniques is comprehensive, facilitating reproducibility. To enhance rigor, provide additional specifics: the supplier and exact properties of lycopodium spores, surface roughness of the steel strips (e.g., root-mean-square value), and calibration methods for tilt angles. Include error propagation analyses for velocity extractions and consider sharing raw data or analysis scripts in supplementary materials.

Thanks for the suggestion. In order to improve the rigor of the manuscript, we added the requested experimental details in the main text.

In addition, we added in the SI an example of extraction of the velocity from the drop trajectory (new figure S6). Owing to the high frequency of data acquisition, uncertainties in the velocity coming from error propagation are found to be strongly limited. We added accordingly error bars in our figures. We also provide in the SI the code used for our data treatment.

4. Figure 1: The panels clearly illustrate the marble's static and dynamic states. Improve the caption by adding quantitative parameters (e.g., "Drop volume Ω = 5 µL, viscosity η = 1000 mPa·s"). Ensure scale bars are present in both subfigures and label the contact radius λ_o directly on the image. Increase image resolution to prevent pixelation in the final submission.

Good suggestions! We corrected the figure accordingly, and now provide photographs of better quality.

5. Figure 2: The trajectories in panel (a) effectively contrast stationary and oscillatory motions, while panel (b) highlights deviations from the Mahadevan-Pomeau model. Add error bars to velocities in (b) to quantify measurement uncertainty. Specify the fitted equation for the dotted Mahadevan-Pomeau line in the caption or methods. Integrate references to supplementary Movie 1 more prominently, ensuring movies include annotations like timestamps and scale indicators.

Thank you for these remarks. We added the measurement uncertainty in figure 2b. The caption has been modified so that the MP law appears explicitly. In addition, we highlighted the reference to Movie 1 and reported all movie parameters in the SI (final part).

6. Figure 3: The normalization and scaling analyses in panels (a) and (b) robustly support the dynamic contact reduction hypothesis. Replace the color gradient (warm to cold for angles) with a discrete legend for improved accessibility. Justify numerical coefficients in scaling fits (e.g., 0.9, 0.7) more explicitly in the text. Add a schematic inset depicting the four contact regimes to aid comprehension.

Thank you for pointing out this lack of clarity. We have modified figure 3 so that the legend for the angles now appears clearly, with the discrete legend suggested by the referee. In order to facilitate understanding, we also added a definition of the four regimes of contact discussed in the paper, and we clarified the caption by specifying the corresponding prefactors. In addition, we switched the figures for making more logical the presentation, and, more importantly, extensively reworded the text prior to the figure, and following the figure. This section of the paper is key, and it was indeed useful to improve its clarity and fluidity.

7. Figure 4: The derived t^{1/3} scaling for gravitational spreading is innovative; provide a quantitative comparison to wetting drop literature (e.g., reference [12]). In panel (b), bolster the linear fit for α_c versus

R/a with additional data points at smaller R values. Standardize axis labels (e.g., consistently use "R/a") and include units throughout.

Thank you for asking for these details. 1) We provide the requested comparison, which is useful to show that this new spreading law is indeed slow compared to 'usual' wetting dynamics, which eventually explains the dynamical nature of the contacts of the rolling marbles. 2) We put a lot of effort to satisfy the second request of the referee. It is extremely challenging to achieve tiny marbles, so that adding data at small R was also especially challenging. However, we could manage to obtain one additional data point for a marble of 0.75 µL (first point in the data). We have also indicated uncertainty measurements. 3) The plot in figure 4b is made with *R/a* (ratio between drop radius and capillary length) in abscissa, which has no units. 4) More importantly, we added a new curve (figure S3 in the SI) that shows that liquid pearls (viscous non-wetting drops on water repellent materials) follow the same gravitational spreading law as marble, which emphasizes the generality of this law.

8. Figures 5 & 6: Those time-lapse shots really bring the "acrobatics" to life—key to the story. Jazz them up with velocity arrows or outlines on each frame. In Figure 5c, mark the regimes (like disk, torus) right on the plot. For Figure 6b, include a simple cycle diagram to clarify the transitions. Ensure the supplements (S1–S3 and Movies 3–4) are consistent, maintaining the same slowdown rates and scale bars.

Thank you for these useful suggestions. 1) We added a new plot in the SI (figure S6), which recaps all successive regimes of velocity during the acrobatics. 2) We marked the regimes (disk/torus) in figure 5c. 3) We clarified the figure 6b by naming the different shapes met during the marble transformations. 4) The scales of the supplementary figures are not all identical because their purpose is to zoom in on specific details and transitions. Regarding the videos, the scale bars and slowdown factors are identical. 5) The acrobatics being indeed key in our story, we added two more examples of this phenomenon, that is, nearly-identical sequences at different marble coverages and liquid viscosities (figures S5 and S7) – showing the robustness of the phenomenon.

9. The Mahadevan-Pomeau model extensions and scaling arguments are logical, but the l(V) derivation should be further developed using lubrication theory or dimensional analysis. Note limitations, such as powder coating effects on dissipation; recommend testing different grain sizes. Calculate Reynolds numbers for all velocities to confirm the low-Re assumption.

Thanks for the suggestions. 1) We introduce the capillary number earlier in the text (last paragraph on page 4), which is useful to anticipate that shape deformations might occur in our experiments if the velocity is large enough to make this number larger than unity. Later (second paragraph of page 7), we confirm that the dynamic contact we model can be expressed as a function of the capillary number, and deviates from the quasi-static limit (MP model) when Ca exceeds a value of order unity. 2) We added an experiment in the SI comparing the acrobatic regime for different grain sizes and different surface grain densities (figure S5). This experiment shows that these parameters do not affect the drop dynamics. We believe that dissipation due to the coating is negligible at high velocity, which explains why the dynamics remains identical regardless of the grain properties. 3) We have also detailed the calculation of the Reynolds number for the different regimes in the revised manuscript (last paragraph on page 4), thereby justifying the low-Reynolds-number assumption.

10. The paradoxical viscosity effect is well explained, but its practical uses—such as improving the movement of viscous droplets in pharmaceutical delivery—should be expanded on. It's also essential to highlight future research directions, such as investigating intermediate viscosities, exploring non-Newtonian fluids, or studying uncoated pearls. Additionally, references should be updated to include recent studies on liquid marbles published after 2020 to ensure the discussion is comprehensive.

We followed the referee's advices and expanded the future research directions in our conclusion, and introduced recent references that discuss developments in the field and potential applications.

11. The tenfold increase in velocity and related instabilities present original findings that challenge current paradigms with measurable data. A clearer comparison to other non-wetting systems and supporting data for the "tenfold" claim would strengthen the work. Exploring scalability and practical applications could further enhance its real-world relevance.

We clarified this point. 1) We simplified the paragraph introducing our finding (end of page 2), without mentioning a tenfold increase not yet observed at this point of the paper. 2) In paragraph 3 of page 4, we define more clearly what we mean by “tenfold”, which is the comparison between our measurements and the expectation from the MP model. 3) As mentioned in point 7, we added a figure about the original spreading behaviour for another non-wetting system (figure S3 in the SI).

Referee 3

1. The paper reports the viscosity-driven phenomena. Perhaps, the explicit calculation of the Reynolds and capillary numbers will be useful for a reader.

Thank you for this useful suggestion. We strengthened the discussion regarding the Reynolds number and we introduced an additional discussion about the capillary number (last paragraph on page 4). See also the beginning of the second paragraph on page 7, where it is quite enlightening to re-introduce the capillary number.

2. The viscosity is strongly dependent on temperature; thus, the experiments carried out under different temperatures are strongly recommended.

Thank you for this remark. We have added a new experiment to illustrate the effect of viscosity on the observed phenomenon (Figure S7 in the SI). It shows that the phenomenon hardly depends on viscosity, with an average velocity and shape transformations that remains essentially unchanged. We varied the viscosity between 800 and 1400 mPa.s, corresponding to a significant change in temperature between 26°C and 20°C (much larger than the variation of temperature in our experiments).

3. Does the reported effect depend on the coating of the viscous marbles?

Thank you for this relevant question. We performed new experiments where we vary both the density of grains at the surface of a marble, and their size. We do not observe any difference in the dynamics of these marbles, and we added a new supplementary figure (figure S5) where we report chronophotographs of marbles either “classical”, that is, coated with lycopodium grains and a surface packing fraction of 0.8, or coated with the same grains yet dilute (packing fraction of 0.5), or with smaller grains (with diameter of 4 μm instead of 30 μm).

4. Perhaps, the paper:On the Nature of the Friction between Nonstick Droplets and Solid Substrates, Langmuir 2010, 26, 15, 12479–12482, will be useful for the authors.

This paper is interesting but slightly out of the scope of our paper where we are far above the critical angles for moving marbles. Instead, we chose to cite a comprehensive review by the same author – that includes many generalities about marbles, and which is indeed very relevant as a reference.

We would like to thank the reviewers for carefully reading the revised paper. We are glad they ound the paper worth being published in *Nature Communications*.
